# Goodhill syndrome: a case report

**DOI:** 10.11604/pamj.2024.47.168.43187

**Published:** 2024-04-05

**Authors:** Anouar Ben Ameur El Youbi, Meryeme Alami, Najib Benmansour, Mohamed Noureddine EL Alami El Amine

**Affiliations:** 1 Department of Otolaryngology and Cervicofacial Surgery, Hassan II University Hospital, Fes, Morocco

**Keywords:** Goodhill syndrome, fixed malleus head, middle ear, otology, case report

## Abstract

Conductive hearing loss with a normal tympanic membrane is a common reason for otolaryngology consultation, with otospongiosis being the most frequent cause and House syndrome being extremely rare, requiring systematic investigation. We report the case of a 31-year-old woman who presented with conductive hearing loss with a normal tympanic membrane. A temporal bone computed tomography (CT) scan confirmed a House-Goodhill syndrome due to fixation of the malleus head. Surgical intervention was considered to remove the attic bone synostosis with the malleus head, resulting in a significant clinical improvement. The Goodhill syndrome is a rare condition that causes hearing loss with a normal eardrum. The surgery can highly improve the hearing function.

## Introduction

Goodhill syndrome, also known as fixed malleus head syndrome, is a rare entity characterized by the primary idiopathic fixation of the malleus head to the wall of the attic [1]. It poses a significant challenge to the surgeon. A congenital origin is the most likely hypothesis [2,3]. Computed tomography (CT), with its multiplanar reconstructions, provides in-depth analysis of the ear, allowing for the exclusion or confirmation of other differential diagnoses [4].

We present the case of a 31-year-old woman with Goodhill syndrome who underwent release of the fixation of the hammer head.

## Patient and observation

**Patient information:** a 31-year-old female patient, with no significant medical history, presents with unilateral hearing loss progressing over the past 3 years, without a history of recurrent otitis or trauma.

**Figure 1:**
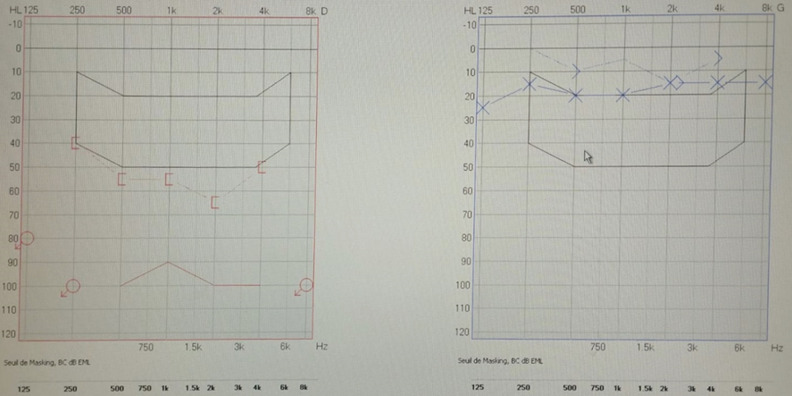
tonal audiometry showing a mixed hearing loss in the right ear with an average loss of 96 dB

**Clinical finding:** on otoscopic examination, the tympanic membranes appear normal. The otolaryngology examination was unremarkable. The hearing loss was confirmed in tonal audiometry. The hearing loss had progressed over the past 3 years, a preoperative tonal audiometry revealed mixed hearing loss in the right ear with an average loss of 96 decibels (dB) (Figure 1).

**Figure 2:**
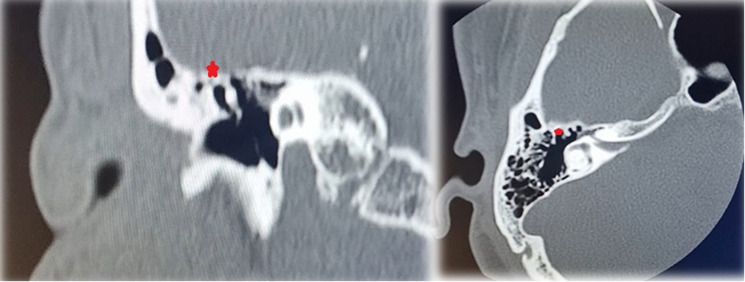
temporal bone CT scan: coronal view threw the anterior portion of the temporal bone on the right, and axial view threw the superior portion of the temporal bone on the left of the figure, the two images show ossification between the malleus head and the tegmen tympani (asterixis)

**Diagnostic and assessment:** radiological assessment was requested. The temporal bone CT scan reveals a bony bridge connecting the superior wall of the tegmen tympani to the hammer head; furthermore, there is no associated otospongiosis noted (Figure 2).

**Therapeutic intervention:** the patient undergoes surgery, and the diagnosis of Goodhill syndrome is confirmed by the presence of a bony attic bridge with the hammer head. It is released without any other specific procedure due to the absence of associated ossicular ankylosis.

**Figure 3:**
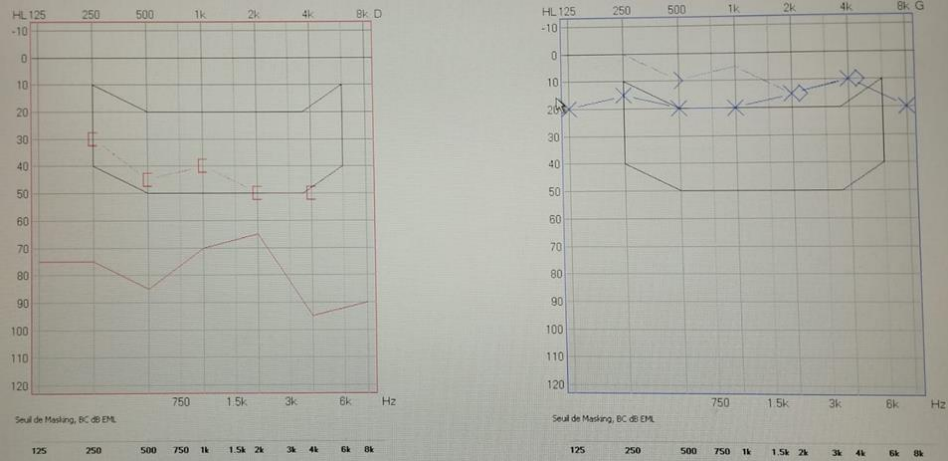
tonal audiometry showing mixed hearing loss with an average loss of 60 dB in the right ear

**Follow-up and outcomes:** after 3 months, the hearing acuity has improved compared to the preoperative assessment, which was confirmed by audiometry (Figure 3). It is planned to regularly review this patient to screen for any potential recurrence.

**Patient perspective:** *“I was handicapped by my deafness, which I had suffered from for a long time. Being around my family and children made me anxious because I had to focus harder and harder to understand what they were saying. I noticed a difference the day following the procedure, and a week later, after removing the otowik, the difference was significant. I am thrilled with the outcome and want to express my gratitude to my surgeon as well as the entire medical and paramedical team for their excellent care”*.

**Figure 4:**
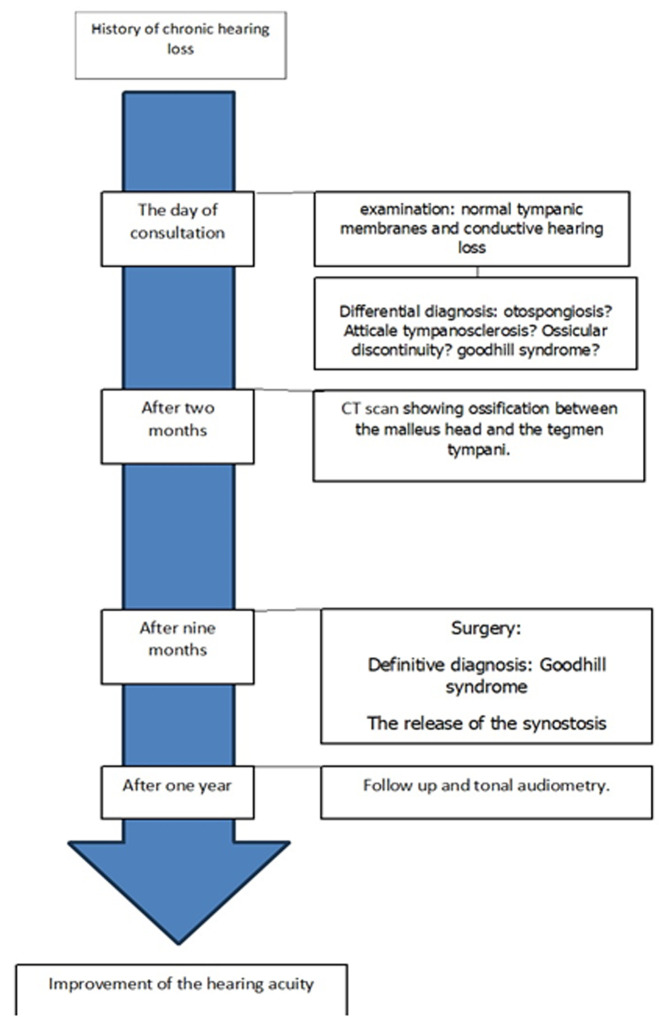
timeline of disease from the occurrence to the hearing improvement

**Timeline of events:** the timeline of events is illustrated in Figure 4.

**Informed consent:** the patient gave his approval for the publication.

## Discussion

Fixed malleus head syndrome encompasses all clinical and radiological manifestations resulting from ossification of the anterior or superior ligaments of the malleus [5]. This creates a bony bridge connecting the malleus to the roof of the attic, this synostosis can occur unilaterally or bilaterally [5]. Initially described by Toynbee in 1860 [6], isolated idiopathic fixation of the malleus head was detailed by Goodhill in 1966 [7]. This phenomenon manifests in a normal middle ear, without a history of otitis or trauma [6].

Its prevalence among cases of conductive hearing loss is estimated to be around 1% [5]. This syndrome typically affects older individuals [6], impacting both sexes without particular preference [8]. Clinically, it is characterized by progressive conductive hearing loss, whether unilateral or bilateral, accompanied by a normal appearance of the tympanic membrane [8].

Several etiopathogenic hypotheses have been described, among which congenital origin is considered the most probable [2,3]. Some authors suggest that synostosis results from a failure of separation between the malleus head and the attic wall during embryogenesis [8]. Others believe that an abnormally elevated position of the malleus head could promote the formation of a bony bridge with the attic wall [9]. According to Moon CN *et al*. this syndrome arises from ossification of the superior or anterior ligament of the malleus, a process associated with aging [6].

Computed tomography plays an important role in visualizing the bony bridge connecting the malleus head to the attic. It is also useful for detecting associated otospongiosis foci, analyzing ossicles, and confirming the absence of facial nerve or inner ear pathway anomalies that may interfere with surgical intervention. Its systematic use is essential to exclude other adjacent pathologies that may alter the therapeutic approach [4].

It is necessary to consider differential diagnosis with other processes of secondary ossification of the middle ear, notably otospongiosis [6], which may present an association with Goodhill syndrome at a rate ranging from 0.6% to 4% [1]. However, confirming the diagnosis remains a peroperative process. The bridge connecting the malleus head may have a bony or fibrous composition. This fixation can occur on the anterior, superior, lateral, or more rarely on the medial wall of the attic [1].

Treatment of Goodhill syndrome aims to restore normal ossicular mobility. It generally involves a type II ossiculoplasty: classical transposition of the incus with a section of the malleus neck. In cases of isolated fixation, such as in our patient, a simpler approach involves directly removing the attic synostosis while preserving ossicular continuity [10].

In our case, once the synostosis was removed the mobility of the ossicular chain was reestablished, so we preferred the second surgical aptitude to avoid the ossiculoplasty to the patient.

## Conclusion

Goodhill syndrome, a rare entity, poses a significant challenge to the surgeon, requiring in-depth knowledge to be identified and suspected in cases of progressive conductive hearing loss. Computed tomography remains the diagnostic modality of choice. The therapeutic approach involves surgical release of the bony attic synostosis with the malleus head.
